# Comparison of sodium content of meals served by independent takeaways using standard versus reduced holed salt shakers: cross-sectional study

**DOI:** 10.1186/s12966-016-0429-z

**Published:** 2016-09-26

**Authors:** Louis Goffe, Frances Hillier-Brown, Aoife Doherty, Wendy Wrieden, Amelia A. Lake, Vera Araujo-Soares, Carolyn Summerbell, Martin White, Ashley J. Adamson, Jean Adams

**Affiliations:** 1 Institute of Health & Society, Newcastle University, Baddiley-Clark Building, Medical School, Newcastle upon Tyne, NE2 4HH UK; 2 Fuse – the Centre for Translational Research in Public Health, Newcastle upon Tyne, UK; 3 Obesity Research Group, School of Medicine, Pharmacy & Health, Wolfson Research Institute, Durham University, Queen’s Campus, Stockton on Tees, TS17 6BH UK; 4 Centre for Public Policy & Health, School of Medicine, Pharmacy & Health, Wolfson Research Institute, Durham University, Queen’s Campus, Stockton on Tees, TS17 6BH UK; 5 Centre for Diet and Activity Research, MRC Epidemiology Unit, University of Cambridge School of Clinical Medicine, Box 285 Institute of Metabolic Science, Cambridge Biomedical Campus, Cambridge, CB2 0QQ UK

**Keywords:** Salt, Sodium, Takeaway, Public health, Diet, Nutrition

## Abstract

**Background:**

Takeaway food has a relatively poor nutritional profile. Providing takeaway outlets with reduced-holed salt shakers is one method thought to reduce salt use in takeaways, but effects have not been formally tested. We aimed to determine if there was a difference in sodium content of standard fish and chip meals served by Fish & Chip Shops that use standard (17 holes) versus reduced-holed (5 holes) salt shakers, taking advantage of natural variations in salt shakers used.

**Methods:**

We conducted a cross-sectional study of all Fish & Chip Shops in two local government areas (*n* = 65), where servers added salt to meals as standard practice, and salt shaker used could be identified (*n* = 61). Standard fish and chip meals were purchased from each shop by incognito researchers and the purchase price and type of salt shaker used noted. Sodium content of full meals and their component parts (fish, chips, and fish batter) was determined using flame photometry. Differences in absolute and relative sodium content of meals and component parts between shops using reduced-holed versus standard salt-shakers were compared using linear regression before and after adjustment for purchase price and area.

**Results:**

Reduced-holed salt shakers were used in 29 of 61 (47.5 %) included shops. There was no difference in absolute sodium content of meals purchased from shops using standard versus reduced-holed shakers (mean = 1147 mg [equivalent to 2.9 g salt]; SD = 424 mg; *p* > 0.05). Relative sodium content was significantly lower in meals from shops using reduced-holed (mean = 142.5 mg/100 g [equivalent to 0.4 g salt/100 g]; SD = 39.0 mg/100 g) versus standard shakers (mean = 182.0 mg/100 g; [equivalent to 0.5 g salt/100 g]; SD = 68.3 mg/100 g; *p* = 0.008). This was driven by differences in the sodium content of chips and was extinguished by adjustment for purchase price and area. Price was inversely associated with relative sodium content (*p* < 0.05).

**Conclusions:**

Using reduced-holed salt shakers in Fish & Chip Shops is associated with lower relative sodium content of fish and chip meals. This is driven by differences in sodium content of chips, making our results relevant to the wide range of takeaways serving chips. Shops serving higher priced meals, which may reflect a more affluent customer base, may be more likely to use reduced-holed shakers.

## Background

Takeaway food consumption makes significant contributions to total dietary intake [1]. Emerging evidence of associations between takeaway food consumption and both total diet [1], and body weight [2], has led to public health action to improve the nutritional quality of takeaway food [3, 4]. One particular area of focus has been dietary salt (sodium chloride, or simply ‘salt’) reduction [3]. Single takeaway meals frequently contain more salt than the World Health Organization’s maximum recommended daily intake for adults of 5 g [5–9]. In systematic reviews, reductions in salt intake have been associated with reduced blood pressure [10–12]; and higher blood pressure with stroke and ischaemic heart disease events and mortality [13, 14].

In the UK, traditional Fish & Chip Shops, serving a core offering of battered and deep-fried white fish with chipped and deep-fried potatoes, account for up to one-third of takeaways.^1^ Traditionally in Fish & Chip Shops, hot food is served into disposable packaging, seasoning (including salt as a minimum) offered and added by the server, and food wrapped – all in front of the customer. The addition of server-added ‘discretionary’ salt is relatively unique to these settings. In this context we use the term ‘discretionary’ salt, to mean salt that is added after food has been prepared but before consumption.

Providing outlets with reduced-holed salt shakers is one method that has been used to reduce salt use in UK takeaways. Building on observational findings that discretionary salt use is related to the size and number of holes in salt shakers [15], standard shakers with 17 holes are replaced with equivalent ones with 5 holes [3]. In a number of documented cases, individuals working for or with local authority environmental and public health departments have offered takeaway outlets reduced-hole salt shakers free of charge [16]. These shakers can also be purchased by outlets directly from wholesalers. We do not have good information on uptake of these shakers across the board (although the current work documents uptake in the areas studied), or factors influencing uptake, nor have the effects of these shakers on the salt content of food served been formally tested.

**Fig. 1:**
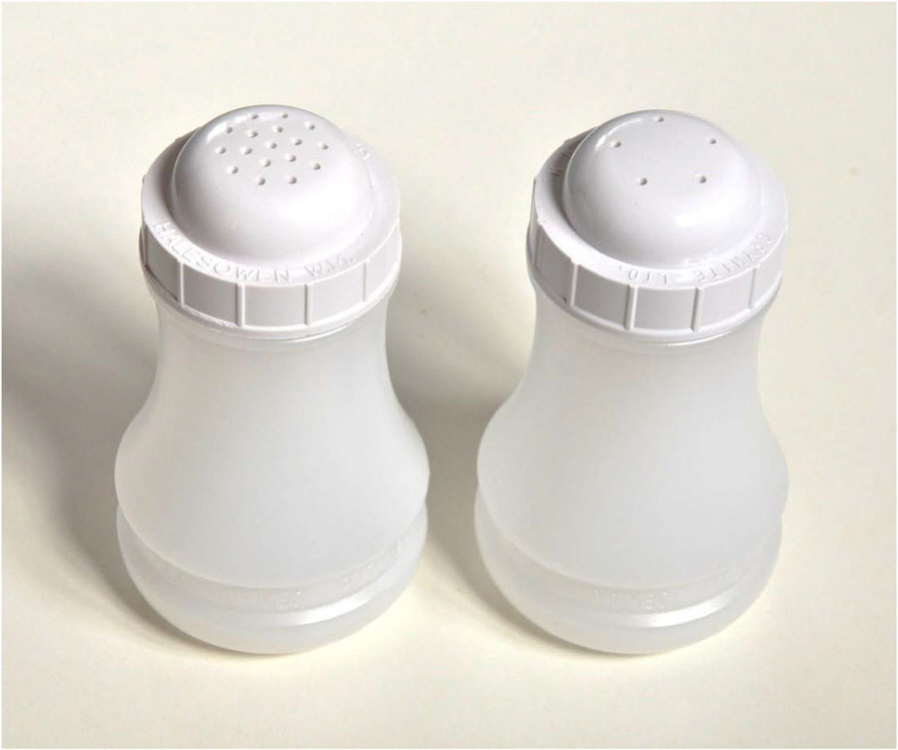
17 (*left*) and five (*right*) holed salt shakers. Foodnote: Image credit: Martin White © 2015

Five-holed salt shakers (5HSS) are relatively cheap (~£2.50; $3.54; €3.14) and comparable in price, look and feel to 17-holed salt shakers (17HSS; see Fig. 1). The ‘health-by-stealth’ approach of 5HSS is particularly attractive and acceptable to both public health practitioners and takeaway managers and staff [3, 17]. Whilst 5HSS have been particularly associated with Fish & Chip Shops, their use has been encouraged across the takeaway sector for both servers and customers [3]. Although we are not aware of 5HSS being used outside of the UK, they may be appropriate elsewhere.

In controlled settings, we found that 5HSS delivered around one-third of the salt of 17-holed salt shakers (17HSS) [18]. This difference may not translate into practice because, for example, servers might shake for longer with 5HSS than 17HSS [19], or customers ask for additional salt when 5HSS are used [16]. We aimed to determine if there were differences in the sodium content of meals served by Fish & Chip Shops using standard (17HSS) versus reduced-holed (5HSS) salt shakers, taking advantage of natural variations (i.e. not researcher-influenced) in salt shakers used.

## Methods

We conducted a cross-sectional study in two local government areas in northern England in May-June 2015. The populations of both areas are concentrated in medium-sized towns (population sizes 120,000 and 83,000) and rank in the more socio-economically deprived half of all such areas in England.

### Data collection

We took a pragmatic approach to sample size determination and aimed to conduct a census of all Fish & Chip Shops in the two study areas. To identify Fish & Chip Shops, we conducted text analysis of a national database of food businesses kept to administer statutory food hygiene inspections (www.ratings.food.gov.uk). We searched business names for those likely to be Fish & Chip Shops (e.g. those containing ‘fish’, ‘fry’, ‘chips’ and derivatives). Additional Fish & Chip Shops identified in the study areas during fieldwork were added to the sample.

In each shop, a researcher (LG in area 1 and FHB in area 2) ordered and purchased one standard fish and chip meal. Researchers remained incognito (i.e. did not identify themselves to servers or customers as researchers). Researchers accepted any salt offered by the server, but did not indicate how much they wanted. Researchers noted the type of salt shaker used and meal price and recorded these soon after leaving shops.

Meals were stored in their packaging in insulated bags for transport to the laboratory. Here they were disaggregated into their components parts of fish, chips, and fish batter, weighed using scales accurate to 0.1 g (MyWeigh, i2600) and frozen at −18 °C in polythene bags until analysis. Any ‘loose’ salt that was contained in packaging but had not ‘stuck’ to food was likely to have been transferred with food and included in the chips component.

### Sample analysis

Sodium was measured in thawed, homogenised and desiccated samples using flame photometry (Jenway, PFP7) in October-December 2016 [20]. Due to resource constraints, analyses were completed in singlicate only. Sodium values were converted to salt values by multiplying by 2.542 [21].

### Data analysis

We compared the absolute and relative (per 100 g) sodium content of meals served by shops using 5HSS vs 17HSS using linear regression. Here, sodium content was the outcome and salt shaker the exposure. Separate analyses were conducted for full meals as well as component parts (i.e. fish, chips and fish batter). In addition to unadjusted analyses, analyses adjusted for the potential confounders of local government area and meal price were conducted. As a male researcher (LG) collected data in area 1 and a female researcher (FHB) data in area 2, adjustment for area also served to adjust for researcher gender.

## Results

Sixty-two shops were identified from the food hygiene database. Five of these were permanently closed on visiting, whilst eight additional shops were identified during fieldwork. Thus, sample meals were purchased from 65 shops. Samples from four shops were excluded due to no server-added salt being offered (*n* = 2) and uncertainty about the type of salt shaker used (*n* = 2). Of the remaining 61 shops, 29 (47.5 %) used 5HSS and 32 used 17HSS.

Descriptive information and unadjusted results are summarised in Table 1. Mean (standard deviation; SD) absolute sodium content of all meals was 1147 mg (424 mg) – equivalent to 2.9 g of salt. Mean (SD) total meal weight was 724 g (145 g). Post-hoc t-tests revealed no difference in the weight of meals, or their component parts, by type of salt shaker (ps > 0.05). In unadjusted regression analyses, there were no statistically significant differences in absolute sodium content of total, or component parts of meals purchased from shops using 5HSS vs 17HSS.

**Table 1:** Unadjusted comparison of sodium in standard fish & chip meals from shops using 5 vs 17-holed salt shakers

	Mean (SD) weight (g)	Mean (SD) sodium (mg)			Mean sodium in 5HSS as % of 17HSS	Unadjusted regression analysis of 5HSS compared to 17HSS; β (95 % CI)
		All (*n* = 61)	17HSS (*n* = 32)	5HSS (*n* = 29)		
Total sodium						
Fish	161.9 (40.3)	357.1 (156.1)	352.2 (162.7)	362.4 (151.1)	102.9	10.2 (−70.5 to 90.9)
Chips	437.7 (101.8)	460.5 (296.7)	526.6 (366.4)	387.5 (171.6)	73.6	−139.1 (−288.2 to 10.1)
Batter	122.2 (37.9)	329.8 (171.5)	355.9 (192.8)	300.9 (142.1)	84.5	−55.0 (−142.6 to 32.5)
Meal	724.4 (145.2)	1147.3 (423.7)	1234.8 (493.9)	1050.9 (310.3)	85.1	−183.9 (−397.8 to 30.1)
Sodium per 100 g						
Fish	NA	227.9 (95.4)	231.2 (91.0)	224.1 (101.6)	96.9	4.2 (−45.8 to 54.1)
Chips	NA	107.8 (64.4)	127.5 (77.0)	86.1 (37.2)	67.5	−40.7 (−73.6 to −7.8)*
Batter	NA	270.0 (107.8)	288.3 (125.4)	249.9 (81.8)	86.7	−53,2 (−109.6 to 3.1)
Meal	NA	163.2 (59.3)	182.0 (68.3)	142.5 (39.0)	78.3	−37.0 (−66.0 to −8.1)*

*SD* standard deviation, *5HSS* 5-holed salt shaker, *17HSS* 17-holed salt shaker, *CI* confidence intervals

*Statistically significant at *p* < 0.05

Mean (SD) relative sodium content of meals was 163 mg (59) per 100 g – equivalent to 0.4 g of salt per 100 g. In unadjusted analyses, relative sodium content was significantly lower in meals purchased from shops using 5HSS vs 17HSS. Meals from shops using 5HSS contained around 40 mg per 100 g (equivalent to 0.1 g per 100 g of salt), or 22 %, less sodium than meals from shops using 17HSS. This difference appeared to be attributable to differences in relative sodium content of chips. Chips from shops using 5HSS contained around 42 mg per 100 g (equivalent to 0.1 g per 100 g of salt), or 32 %, less sodium than chips from shops using 17HSS.

Analyses adjusted for meal price and local government area are shown in Table 2. There remained no difference in total sodium content of meals, or their component parts, after adjustment. Neither meal price nor area was associated with total sodium content in any analysis.

**Table 2:** Adjusted comparison of sodium in standard fish & chip meals from shops using 5 vs 17-holed salt shakers

	Adjusted linear regression analysis of 5HSS compared to 17HSS; β (95 % CI)			
	Fish	Chips	Batter	Meal
Total sodium				
Sodium (mg); 5HSS compared to 17HSS	8.4 (−77.5 to 94.2)	−126.0 (−280.5 to 28.4)	−76.9 (−168.3 to 14.6)	−194.5 (−421.6 to 32.6)
Meal price (£)	31.3 (−50.6 to 113.2)	−141.6 (−288.9 to 5.8)	33.1 (−54.1 to 120.3)	−77.2 (−293.9 to 139.4)
Area (2 vs 1)	−5.1 (−94.8 to 84.5)	−31.9 (−193.2 to 129.3)	−51.5 (−147.0 to 44.0)	−88.6 (−325.7 to 148.6)
Sodium per 100 g				
Sodium per 100 g (mg); 5HSS compared to 17HSS	21.0 (−29.7 to 71.7)	−32.0 (−65.0 to 1.0)	−41.2 (−100.3 to 17.9)	−28.0 (−57.2 to 1.2)
Meal price (£)	−20.5 (−68.9 to 27.8)	−41.4 (−72.8 to −9.9)*	−38.8 (−95.1 to 17.6)	−31.2 (−56.1 to −3.4)*
Area (2 vs 1)	40.3 (−12.7 to 93.3)	6.6 (−27.9 to 41.0)	28.6 (−33.1 to 90.3)	12.5 (−18.0 to 42.9)

*5HSS* 5-holed salt shaker, *17HSS* 17-holed salt shaker, *CI* confidence intervals

*Statistically significant at *p* < 0.05

Adjustment for meal price and area extinguished the relationship between shaker type and relative sodium content of meals and chip components. Meal price was also significantly inversely associated with relative sodium content of both meals, and chip components.

## Discussion

### Summary of results

This is the first study we are aware of to determine whether using reduced-holed salt shakers is associated with lower sodium content of takeaway meals. We found that standard fish and chip meals purchased from Fish & Chip Shops using 5HSS had significantly lower relative sodium content than those purchased from shops using 17HSS. This appeared to be driven by a difference in relative sodium content of the chips component of meals and was extinguished by adjustment for area and meal price – higher cost meals had lower relative sodium content. There was no difference in absolute salt content of meals, or component parts of meals, purchased from shops using 5HSS vs 17HSS.

### Interpretation and implications of findings

The total relative sodium content of meals in the sample was comparable to that in fish and chip meals reported in a previous survey conducted in a different part of England [8]. In this previous work, 51 portions of fish and chips were bought in a large urban conurbation in the North West of England. Mean absolute salt content per meal was 3.00 g (equivalent to 1181 mg of sodium and comparable to the 1147 mg found in the current work – see Table 1) and mean relative salt content was 0.43 g/100 g (equivalent to 169 mg of sodium and comparable to the 163 mg found in the current work – see Table 1). Whilst the mean relative salt content we found (0.4 g per 100 g) would be considered ‘medium’ according to UK front-of-pack traffic light labelling [22], the absolute salt content (2.9 g) equates to more than half of the WHO’s maximum recommended daily salt intake for adults [9].

Our findings of unadjusted differences in relative, but not absolute, sodium content by shaker type suggest there may be a systematic difference in total weight of meals served between shops using 5HSS vs 17HSS. Whilst meals from shops using 5HSS weighed a mean of 58 g more than those from shops using 17HSS and this was primarily due to larger chip serving sizes (a mean of 47 g more in shops using 5 versus 17HSS), these differences were not statistically significant. As such, this may also reflect random, rather than systematic, variation.

Our unadjusted results suggest that customers eating full meals would not consume significantly different amounts of salt in meals from shops using 5HSS vs 17HSS. However, customers consuming similar absolute quantities of meals would consume less salt in meals from shops using 5HSS vs 17HSS. Whilst it is clear that people eat more when given larger portions [23], it is not clear how meals from Fish & Chip Shops are eaten and how this varies by overall portion size. Some meals may be eaten in full by a single person, others shared, and others eaten only in part with leftovers discarded. Further work exploring patterns of consumption is required to determine the population impact of our findings.

Differences in relative sodium content of meals from shops using 5HSS vs 17HSS appeared to be due to differences in relative sodium content of the chips component of meals. In a standard fish and chip meal, the chips are likely to have a larger overall surface area than the battered fish – meaning they are more exposed to discretionary salt. Chips may also provide a more adherent surface for salt granules than fish batter. High sodium content of chips may also reflect salting practices – researchers observed that chips were often served and salted first, before the fish was placed on top.

As the chip component of meals had the highest absolute sodium content of meals, salt reductions here have the largest potential to lead to reductions at the meal level. This also makes our results of relevance to the wide range of takeaways – beyond Fish & Chip Shops – in the UK that serve chips with discretionary salt.

Our findings that adjustment for meal price and local government area extinguished the association between salt shaker and relative sodium content, and that meal price was inversely associated with relative sodium content, hints at one potential determinant of salt shaker use. It is possible that those Fish & Chip Shops serving higher priced meals have more affluent customers. As affluence is associated with greater dietary knowledge [24] shops serving these customers may be more willing to use 5HSS. Alternatively, or in addition, total sodium intake decreases with increasing affluence in the UK [25]. More affluent customers may, therefore, have less pronounced taste preferences for salt – driving less salt use by servers in the takeaways these customers frequent. It should be noted, however, that a post-hoc t-test revealed no difference in meal cost between shops using 5HSS vs 17HSS (*p* > 0.05).

The relative sodium content of fish (removed from batter) we found (228 mg/100 g – see Table 1) was much higher than the 100–110 mg/100 g listed for a range of white fish in standard food tables [26]. This suggests that salt has been added during preparation – possibly leaching out of batter. Further work to change the amount of salt added during preparation may be required to achieve substantial reductions in the salt content of meals from Fish & Chip Shops.

### Strengths and limitations of methods

Incognito researchers purchased meals from shops under natural conditions, maximising the likelihood that sample meals were representative of all meals produced by included takeaways. However, there may be unmeasured within-takeaway, between-meal variation in sodium content, leading to error and potentially bias. Variables at the takeaway level that may also have influenced salt content but that we were unable to measure include: server gender and experience, and the length of time shops have been in business and their popularity. Time of day and day of week may also have confounded our results. However, in post-hoc tests, we found no evidence that either varied by salt shaker used (ps < 0.05) or was associated with total absolute or relative salt content of meals (ps > 0.05).

For resource reasons, we only performed sodium analysis in singlicate. As additional repetitions are likely to provide more accurate estimates, this may be a further source of error. Again, there is no reason to believe that this error would vary systematically according to shaker used. Although we did not specifically compare the use of singlicate analyses to performing multiple replications on each sample, the flame photometer was recalibrated using analytical grade sodium chloride diluted in deionised water after every 9–12 samples.

Although there are regional variations in condiments offered in UK Fish & Chip Shops, server-added salt is almost universally offered (as was the case in 97 % of shops in our sample). Our findings are likely to be generalizable across UK Fish & Chip Shops. However, they may not be generalizable to other takeaway types, or takeaways in other countries. Further research is required to confirm the effects of reduce holed salt shakers more widely.

## Conclusions

Meals from shops using reduced-holed salt shakers (5HSS) had lower relative sodium content than those using standard salt shakers (17HSS), but there was no difference in absolute sodium content. Whilst our findings suggest that 5HSS could be a useful public health intervention, additional work will be required to model the likely population impact fraction of 5HSS on total salt intake, blood pressure, and health outcomes such as stroke and cardiovascular disease and hence quantify the health benefits of 5HSS.

The differences in salt content we identified appeared to be particularly driven by differences in the sodium content of chips. This makes the findings of relevance to a wide range of independent takeaways in the UK that serve chips. Differences in relative sodium content were extinguished by adjustment for meal price and area, and there was an inverse association between meal price and relative sodium content. This may reflect and contribute to socio-economic inequalities in diet.

Whilst reduced-holed salt shakers may help reduce ‘discretionary’ salt added after food preparation by servers and consumers, takeaway food appears to be high in salt even before the addition of this discretionary salt. Additional efforts, focusing on salt added during cooking, may be required to substantially reduce the salt content of food served by Fish & Chip Shops and takeaway food more generally.

## Abbreviations

17HSS: 17-holed salt shaker
5HSS: 5-holed salt shaker
SD: standard deviation

## Footnotes

1 Personal communication, E Macguire (2015) – bespoke additional analysis of data described in ref [12].
